# Extensive production of *Neospora caninum *tissue cysts in a carnivorous marsupial succumbing to experimental neosporosis

**DOI:** 10.1186/1297-9716-42-75

**Published:** 2011-06-02

**Authors:** Jessica S King, Bronwyn McAllan, Derek S Spielman, Scott A Lindsay, Lada Hůrková-Hofmannová, Ashlie Hartigan, Sarwat E Al-Qassab, John T Ellis, Jan Šlapeta

**Affiliations:** 1 Faculty of Veterinary Science, University of Sydney, NSW 2006, Australia; 2 Physiology and Bosch Institute, Faculty of Medicine, University of Sydney, NSW 2006, Australia; 3 Department of Pathological Morphology and Parasitology, University of Veterinary and Pharmaceutical Sciences, Brno, 612 42, Czech Republic; 4 School of Medical and Molecular Biosciences, University of Technology, Sydney, NSW 2007, Australia

## Abstract

Experimental infections of *Sminthopsis crassicaudata*, the fat-tailed dunnart, a carnivorous marsupial widely distributed throughout the arid and semi-arid zones of Australia, show that this species can act as an intermediate host for *Neospora caninum*. In contrast to existing models that develop relatively few *N. caninum *tissue cysts, dunnarts offer a new animal model in which active neosporosis is dominated by tissue cyst production. The results provide evidence for a sylvatic life cycle of *N. caninum *in Australia between marsupials and wild dogs. It establishes the foundation for an investigation of the impact and costs of neosporosis to wildlife.

## Introduction

For much of the past 100 000 years Australian fauna have evolved without the presence of the Australian dingo. The dingo mitochondrial (mt)DNA sequences and fossil records indicate that this current top order predator arrived on the continent less than 5000 years ago [1]. Consequently, diseases transmitted by dingoes are exotic to Australian fauna and may present a serious factor contributing to their endangerment. The Australian dingo, together with the domestic dog, is a definitive host for the apicomplexan parasite *Neospora caninum *[2]. Neosporosis is a major protozoal reproductive disease in cattle and a recognised neurological disease in dogs [3-5]. Currently, the majority of research on neosporosis has focused on cattle because the cattle industry identified the disease as a significant economic burden worldwide. However, virtually all vertebrates are assumed to be susceptible to neosporosis, with the degree of pathology varying between host species [4]. Indeed, in other countries, desert dwelling animals such as gerbils appear to be particularly susceptible to this parasite [6-8]. While the presence of tissue cysts determines if the animal can serve as a source of infection for the canid definitive host, tissue cysts are rarely documented in either experimental or naturally infected animals [4,9,10].

In Australia a sylvatic life cycle of *N. caninum *is hypothesised between the dingo and an undefined range of small marsupials and eutherian mammals [5]. The cost to wildlife due to disease transmitted from domestic and farm animals is unknown [11]. Since no information exists on neosporosis in Australian native small marsupials, our aim was to provide evidence using experimentally infected animals. Our trial used *Sminthopsis crassicaudata*, the fat-tailed dunnart, a carnivorous marsupial widely distributed throughout the arid and semi-arid zones of Australia and one of only a few marsupial species bred in laboratory [12,13]. The fat-tailed dunnart (adult body mass 12-16 g) inhabits the same geographical areas as the dingo, feral fox and rangeland cattle, and, as its name suggests, its fat is stored in the tail, from a few millimetres from the base and almost to the tip [12]. This shrew-like marsupial belongs to the family Dasyuridae (Dasyuromorpha; Marsupialia), which also includes other Australian endangered species like mulgaras (*Dasycercus *spp.), quolls (*Dasyurus *spp.), the Tasmanian devil (*Sarcophilus harrisi*) and the little red kaluta (*Dasykaluta rosamondae*) [13].

In this study we show that the fat-tailed dunnart is highly susceptible to neosporosis. It represents a new model for production of *N. caninum *tissue cysts, because numerous cysts of *N. caninum *are present throughout the body of an infected animal, including in the severely affected pancreas with *N. caninum *development confined to the acinar cells.

## Materials and methods

### Tachyzoites of *Neospora caninum*

The isolate used was *N. caninum *NC-Nowra which was isolated from a new born calf in New South Wales, Australia [14]. The tachyzoites of NC-Nowra were maintained and harvested as described previously [2].

### Oocysts of *Neospora caninum*

Oocysts in faecal material were obtained from a naturally shedding dog (dingo hybrid) from an Aboriginal community located in Yuendumu in the Tanami Desert, Northern Territory, Australia. Faeces were kept in 2% potassium dichromate and sporulated oocysts were purified using a standard NaCl flotation technique.

### Experimental animals, experimental trials and monitoring

All animal experiments were approved by the University of Sydney Animal Ethics Committee and complied with NSW Animal Welfare Acts. Experimental *N. caninum *dose regimens were tested on adult male fat-tailed dunnarts, *Sminthopsis crassicaudata*, bred at the University of Sydney (Table 1). Freshly harvested tachyzoites of *N. caninum *were used for intra peritoneal (i.p.) inoculation. Oocysts of *N. caninum *were 4 months old at the time of the feeding experiment (Table 1).

**Table 1:** Summary of experimental *Neospora caninum *infection in the fat-tailed dunnart.

Experimental dose, route	Id	Euthanasia	PCR	cELISA (%I)	IHC
10^4 ^*N. caninum *tachyzoites, i.p.	A1	28 dpi	Neg.^b^	Pos. (74%)	Neg.
	
	A2	28 dpi	Neg.^b^	Pos. (41%)	Neg.
	
	A3	28 dpi	Neg.^b^	Neg. (8%)	Neg.

10^5 ^*N. caninum *tachyzoites, i.p.	B_1_1	16 dpi^a^	Pos.^c^	Pos. (43%)	Pos.
	
	B_1_2	18 dpi^a^	Pos.^c^	Pos. (42%)^f^	Pos.
	
	B_1_3	18 dpi^a^	Pos.^c^	Pos. (51%)^f^	Pos.
	
	B_2_1	13 dpi^a^	Pos.^c^	Neg. (25%)	Pos.
	
	B_2_2	14 dpi^a^	Pos.^c^	Pos. (51%)	Pos.
	
	B_2_3	13 dpi^a^	Pos.^c^	Neg. (19%)	Pos.

20-40 *N. caninum *oocysts, p.o.	E1	46 dpi	Neg.^d^	Neg. (-4%)	Pos.^g^
	
	E2	46 dpi	Pos.^e^	Neg. (4%)^f^	Neg.

^a^animal euthanised due to clinical neosporosis;

^b^DNA isolated from brain, liver and lung tested;

^c^DNA isolated from brain tested;

^d^DNA isolated from brain, liver, spleen and lung tested;

^e^DNA isolated from brain, liver, spleen and lung tested, positive PCR on brain;

^f^sera diluted 1:5 due to limited amount of sera;

^g^IHC positive *N. caninum *stages in spleen (all other organs tested *N. caninum *IHC negative).

To evaluate *N. caninum *infectivity to the fat-tailed dunnart, fifteen 1-2 year old male dunnarts weighing 13.0-18.1 g were housed individually in a controlled light-proof environment with a 12:12-hour light:dark photocycle (Table 1). Every night each animal consumed approximately their own body weight of cat wet food (Mars Pet Food, Australia). Water was provided *ad libitum*. To assess each animal's activity every cage was equipped with an in-house assembled "mouse exercise wheel" with a "cycling odometer reader" attached that recorded running time and trip distance daily during the experiment. Body mass (g) and tail width (mm an index of general health) were monitored throughout the experiment and were analysed by the Mann-Whitney U test (*P *< 0.05 is considered significant). All animals were monitored using a day and night (infrared) surveillance system for activity and, if present, to monitor any adverse responses to the parasite or behavioural change.

Thirteen of the experimental animals were inoculated intraperitoneally using 10^4 ^or 10^5 ^*N. caninum *tachyzoites (Table 1). Animals were inoculated i.p. using *N. caninum *tachyzoites in 0.3 mL of phosphate buffered saline (PBS, pH = 7.2) or PBS only. For each experimental cohort one animal served as a negative uninfected control. An animal immunosuppressed by 3 mg of methylprednisolone acetate administered at the time of *N. caninum *injection (10^4 ^*N. caninum *tachyzoites-experiment A) served as a positive control as suggested previously for a mouse study [10]. Our protocol was designed to increase the inoculation dose incrementally if no adverse clinical signs were observed (starting from 10^4 ^*N. caninum *tachyzoites and increasing by 10x, i.e. 10^5 ^*N. caninum *tachyzoites-experiment B) using new uninfected animals. However, if adverse clinical signs required euthanasia, the experiment was repeated to confirm the finding without increasing the infective dose.

To evaluate infectivity of *N. caninum *oocysts, we purified oocysts from faeces using faecal flotation and fed them to two fat-tailed dunnarts that were monitored as described above. 20-40 oocysts were mixed into each of the animals' daily food; both animals consumed the entire ration overnight.

### Post mortem of experimental animals

All euthanised animals were investigated by full histopathology. Formalin-fixed specimens from the brain, heart, lung, liver, kidneys, eyes, spleen, pancreas, bladder, testes, accessory sex glands, stomach, small and large intestine and skeletal muscle from the thigh were processed to standard paraffin-embedded blocks and 5 μm haematoxylin and eosin stained sections examined. Blood was collected from the heart and separated serum stored at -20°C for later serological assessment.

### Immunohistochemistry for *Neospora caninum*

Immunohistochemistry (IHC) was performed with a goat anti-*Neospora caninum *polyclonal antiserum (210-70-NC, VMRD, Pullman, WA, USA) at a 1:6,000 dilution. Paraffin embedded sections were processed using the Dakocytomation Autostainer system (3% H_2_O_2 _peroxidase block; Proteinase K Ready-to-Use, S3020; Universal LSAB™+ Kit/HRP, K0690; Dako Australia, Campbellfield). To detect *N. caninum *bradyzoites, the polyclonal rabbit antibody anti-*Tg*BAG5 was used at a 1:200 dilution using the IHC protocol described above (rabbit sera were used as control at a 1:200 dilution). The polyclonal rabbit anti-*Tg*BAG5 was kindly provided by Dr Milton McAllister (University of Adelaide, South Australia, Australia). A competitive ELISA for *N. caninum *(VMRD, Pullman, WA, USA) was used to detect *N. caninum *antibodies (Table 1).

### Oil Red O staining for tail fat

Formalin fixed tails of experimental animals were decalcified for four days and cryosections stained with filtered Oil Red O staining for fat (stock solution: 3 mg/mL in isopropanol; working solution: 60% Oil Red O stock solution and 40% distilled water [15]) for 3 h at room temperature and quantified by Image J 1.44i (Wayne Rasband, National Institutes of Health, USA). After correction for background the colour images were segmented using the colour threshold to separate red areas of fat from the skin, muscle and bone; their ratios were calculated to quantify the degree of tail fattening. Data were analysed using the Student's *t*-Test (*P *< 0.05 is considered significant).

### PCR identification of *Neospora caninum*

Nucleic acid from the oocysts was extracted from 200 μL of the upper layer from a salt flotation containing oocysts, using the FastDNA Soil Kit Protocol with a Fast Prep-24 Homogenisation System equipped with QuickPrep Adapter (MP Biomedicals, Australia); the speed was set at 6.0 for 40 s.

From each experimental animal, specimens of the brain, lung, liver and spleen were used for DNA isolation using the ISOLATE Genomic DNA Mini Kit (Bioline, Australia). DNA was isolated from blood using the PureLink DNA Kit (Invitrogen, Australia).

Species-specific PCR targeting the Nc5 locus (Np6+/Np21+primers) was used to determine presence of *N. caninum *DNA [16]. *N. caninum *ITS1 rDNA was amplified using JS4-Tim11 primers as described previously [2,17,18]. The MS10 microsatellite was PCR amplified and sequenced using the following conditions and primers described by Basso et al. [19] and Pedraza-Díaz et al. [20]. Each reaction of 25 μL contained 12.5 μL of 2x SAHARA Mix (BioLine), 0.5 μL of each 10 mM primer, and 100 ng of extracted DNA; deionised sterile water was used as a negative control. PCR was performed in an Eppendorf Mastercycler Personal. PCR products were cloned using the TA-TOPO Cloning Kit (Invitrogen, Australia) according to the manufacturer's instructions. Randomly selected plasmids with target inserts were sequenced by AGRF (Westmead, Australia). Sequences were assembled, aligned with related sequences and analysed using the CLC Main Workbench 5.5 (CLC bio, Denmark). Nucleotide sequence data reported in this paper are available from GenBank™, EMBL and DDBJ databases under the accession numbers [GenBank: HQ873010-HQ873034].

## Results And Discussion

**Figure 1:**
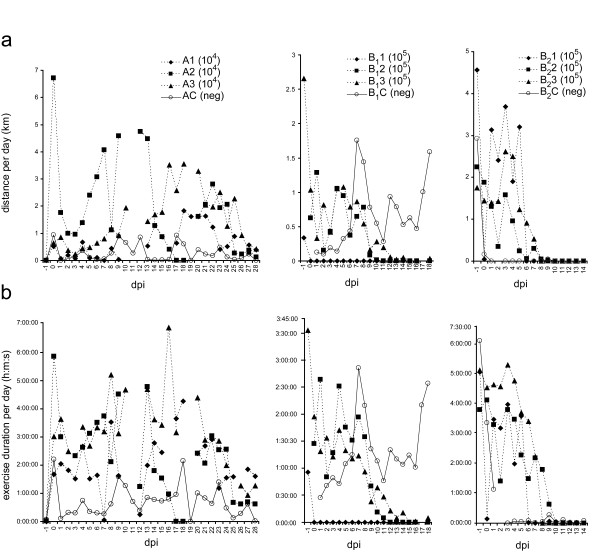
**Daily exercise distance and duration of fat-tailed dunnarts during *N. caninum *tachyzoite infection**. The activity of all experimental animals was individually monitored and is presented as a daily sum; most activity was recorded during the night for all days post-infection. The activity reflects the use of an exercise wheel in their enclosures. Daily number of revolutions is translated into daily distance per animal (a) and time spent on the wheel (b). Experiment A, B_1 _and B_2 _are shown (negative control animals' daily activity [o] is connected with a solid line; *N. caninum *infected animals' daily activities [◆●■] are connected with intermittent lines). B_1_1 and B_2_C failed to use the exercising wheel; B_1_1 remained active (running within the enclosure) until day 8 post infection; B_2_C remained active throughout the experiment.

**Figure 2:**
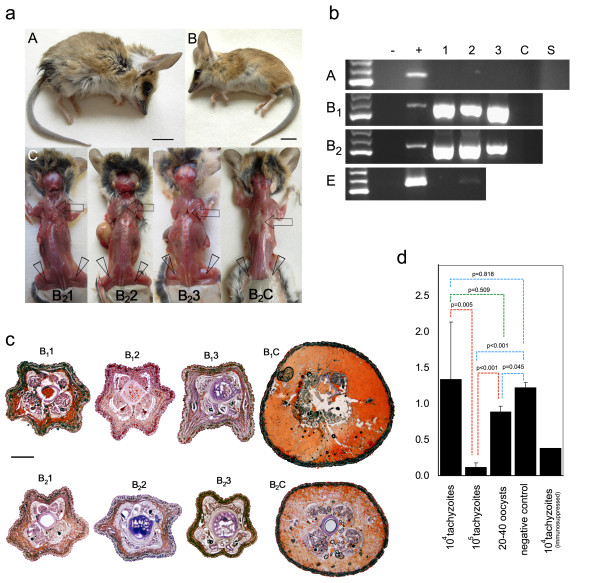
**Neosporosis in fat-tailed dunnarts**. Appearance of the infected dunnarts (a). The fur of infected dunnarts was roughened due to marked incontinence (A), recognised as wet anal, perineal and base of the tail regions (B_2_2). The negative control animal (B_2_C) had clean groomed fur including around the tail base (B). Interscapular (arrow) and gluteal (arrowheads) fat was absent from infected animals (B_2_1, B_2_2, B_2_3) compared to the control animal (B_2_C) which had marked fat deposition at both sites (C). PCR detected presence of *N. caninum *DNA using Np6+/Np21+ primers (b). A *N. caninum *specific reaction appeared as a 330 bp amplicon. In experiment A the infectious dose was 10^4 ^*N. caninum *tachyzoites (1-3); this experiment included a *N. caninum*-infected animal immunosuppressed with methylprednisolone acetate as a control (S) plus an animal not infected with *N. caninum *as a negative control (C). Animals in experiments B_1 _and B_2 _were inoculated with 10^5 ^*N. caninum *tachyzoites (1-3). Animals in experiment E were inoculated orally with less than 20-40 oocysts of *N*. caninum (1, 2). Negative (-, water) and positive (+, *N. caninum *DNA) controls were included for each PCR batch. The left lane is a 100 bp DNA ladder (300, 400, 500 bp). Gel is stained using GelRed and inspected under UV. Oil Red O staining of tails of fat-tailed dunnarts (c) and ImageJ colour threshold applied to separate red areas of fat from the skin, muscle and bone for statistical analysis (d). Tail cross sections of animals in experiments B_1 _and B_2 _(c). Ratio means were compared for different inoculation doses (10^4 ^*N. caninum *tachyzoites, *n *= 3; 10^5 ^*N. caninum *tachyzoites, *n *= 6; < 50 *N. caninum *oocysts, *n *= 2; control animals, *n *= 3) and comparison made using Student's *t*-Test (*P *< 0.05 is considered significant).

A dose of 10^5 ^tachyzoites of *N. caninum *resulted in severe clinical signs, including sudden onset of lethargy and progressive paralysis of the hind limbs, 1-3 days prior to euthanasia in two consecutive experimental trials (B_1_, B_2 _experiments; *n *= 6). Dunnart activity, as measured by the distance run over 24 h (predominantly at night, Figure 1a) and the time spent on the exercise wheel (Figure 1b), was reduced from days 6-10 onwards for all infected animals compared to the control. Incontinence was one marked clinical sign observed one day prior to or on the day of euthanasia, recognised as a wetting around the urogenital sinus region, perineum and base of the tail region (Figure 2a). Negative control animals remained clinically normal (*n *= 2) throughout the experiment. The *N. caninum *infected dunnarts did not consume all their food from day 4 on 45% and 55% of occasions (B_1_: 18/40 days; B_2_: 17/31 days), compared to the negative control animals (B_1_: 0/28 days; B_2_: 1/14 days) that consumed all their food every day (except one) throughout the experiments (Additional file 1). Occasional spontaneous fasting for one day is normal behaviour for this species (BM, unpublished observation). Water intake was not adversely affected and was comparable to the negative control. All inoculated animals were euthanised due to severe clinical signs (Table 1). DNAs isolated from *N. caninum *infected dunnarts' brains were positive for *N. caninum *DNA (Figure 2b).

**Figure 3:**
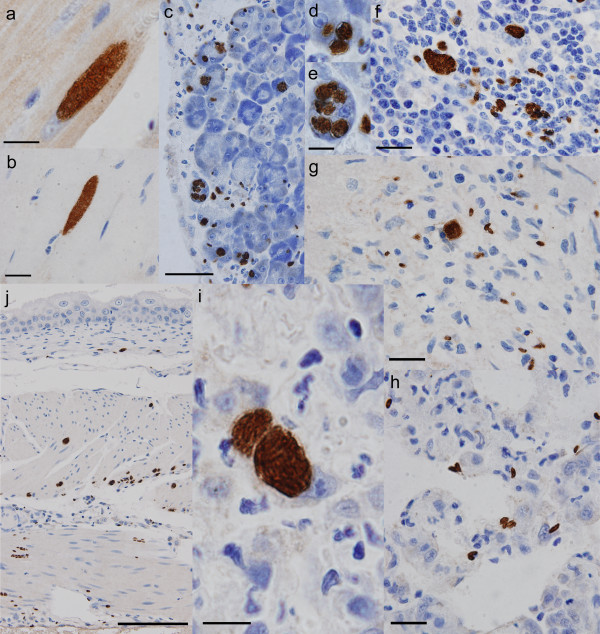
**Immunohistochemical detection of *Neospora caninum *developmental stages in fat-tailed dunnart**. Positive IHC staining with anti-*Neospora *antibodies: staining of elongated tissues cysts of *N. caninum *in cardiac (a) and skeletal muscle (b); pancreas containing a large number of free zoites (c) and multiple round cysts within a single acinar cell (d, e); lymph node (f), brain (g) and lung (h) with scattered developmental stages including free zoites; lung tissue with a large round cyst filled with zoites within a large mononuclear cell likely to be a pulmonary macrophage (i); and a cross-section of the urinary bladder wall with *N. caninum *stages apparent in all layers of the detrusor muscle (j). Tissues from animals inoculated 10^5 ^*N. caninum *tachyzoites (B_1_1: A, H, I; B_2_1: B-G, J). Bars: A, B, F, G, H = 20 μm; C = 50 μm; D+E, I = 10 μm, and J = 100 μm.

**Figure 4:**
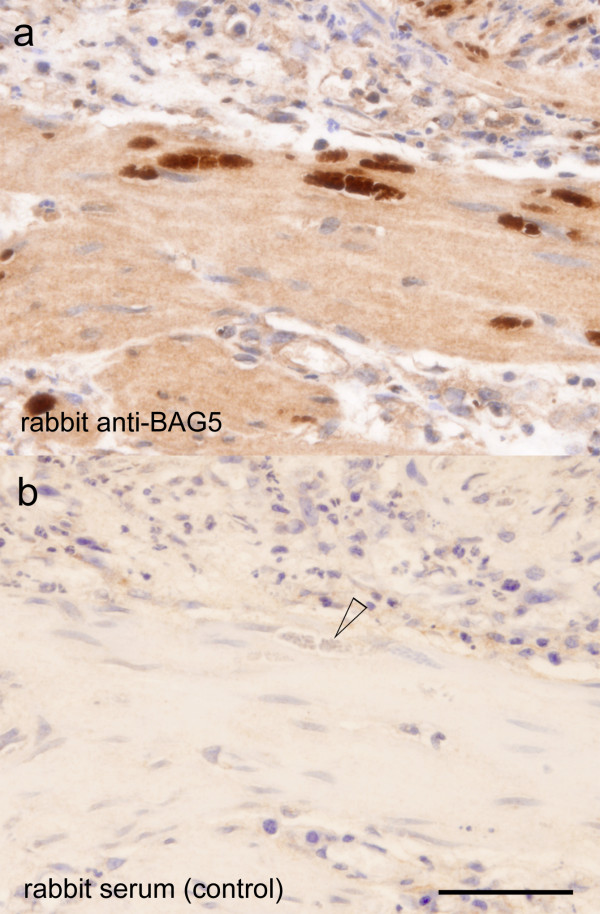
**Detection of bradyzoites using BAG5 immunohistochemistry**. *Neospora caninum*-infected fat-tailed dunnart urinary bladder muscle showing bradyzoites of *N. caninum *labeled with rabbit anti-*Tg*BAG5 (a). A negative control (b) using rabbit serum does not label the cysts of *N. caninum *(arrow). Histological sections are from the same region of the urinary bladder using consecutive sections (animal inoculated 10^5 ^*N. caninum *tachyzoites, B_1_1). Bar, 50 μm.

At post mortem infected animals exhibited substantially less subcutaneous interscapular and hind limb fat compared to the control animal although body mass differences were not significant (Mann-Whitney U test *P *= 0.35, Figure 2a, Additional file 2). However, the tails of infected animals were thinner compared to the negative control animals (Mann-Whitney U test *P *= 0.05; Supplementary Table S2). Oil Red O staining demonstrated significantly (*P *< 0.05) reduced tail fat deposits (Figures 2c and 2d). In experiment B_1_, all animals seroconverted to *N. caninum *(16 and 18 days post-infection (dpi)) as determined using cELISA (Table 1); moreover, sera diluted 1:5 were sufficient for a positive cELISA (> 30% inhibition). The competitive ELISA for *N. caninum *(VMRD) is validated for cattle and goats [21], however its design does not preclude using it for heterologous sera such as that collected from dunnarts. In experiment B_2_, only the dunnart euthanised on 14 dpi had seroconverted to *N. caninum *(51% and 54% inhibition using undiluted and 1:5 diluted sera). Dunnarts euthanised on 13 dpi were negative for *N. caninum *in cELISA (7%/25% and 9%/19% inhibition using undiluted/1:5 dilutes sera, respectively). The timing of seroconversion in this experiment is similar to findings in gerbils (*Meriones unguiculatus*) which seroconverted to *N. caninum *after 13 dpi with *N. caninum *oocysts [22]. In our experimental dunnarts (B_1_), IHC using anti-*Neospora *antibodies confirmed the presence of numerous positively staining free zoites in the heart, lung, pancreas, spleen, mesenteric lymph node, adrenal gland, urinary bladder and skeletal muscle, and multiple cysts of *N. caninum *were found in the heart, lung, pancreas, adrenal gland, urinary bladder and skeletal muscle (animals were euthanised at 16 and 18 dpi; Figures 3a, h and 3i). These *N. caninum *cysts were demonstrated to contain bradyzoites using anti-*Tg*BAG5 (Figure 4). The polyclonal rabbit antiserum directed against surface protein BAG5 (also known as BAG1/hsp30) of *Toxoplasma gondii *is known to cross react with *N. caninum *bradyzoites and cysts [23-25]. Further, several IHC-positive *N. caninum *stages were detected in the brain, gall bladder wall, gastric wall and within the intestinal villi/enterocytes. There was no detectable *N. caninum *observed in the kidney, eye or spinal cord. In the repeat experiment (B_2_), dunnarts euthanised earlier (13-14 dpi) had large numbers of IHC-positive *N. caninum *cysts and free zoites in the pancreas, lymph node, lung, smooth muscle of the urinary bladder and gastrointestinal wall, skeletal muscle, heart and liver (Figures 3b-g and 3j). Fewer *N. caninum *stages were detected in the brain and accessory sex glands. *N. caninum *was not detected in the kidney or eyes. In all cases IHC confirmed the presence of abundant *N. caninum *zoites (2-5 μm, banana-shaped) in most tissues examined. Multiple growing cysts as well as free zoites were present in the heart and striated musculature throughout the body (Figures 3a and Figure 3b), as well as the pancreas (Figure 3c) with distinct multiple multiplying *N. caninum *within acinar cells (Figures 3d and 3e). In particular, the bladder smooth muscle was parasitised with large number of cysts and free zoites (Figure 3j), suggesting impaired bladder function which is consistent with the observed incontinence. Free zoites were the dominant stage detected in the brain (brain stem, Figure 3g).

Histopathology showed mild to very marked changes associated with the presence of *N*. *caninum *in many tissues. The most severely affected tissues with recognisable *N. caninum *cysts were the pancreas with large widespread irregular areas of necrosis, degeneration and numerous degenerating neutrophils (plus few to numerous macrophages and cellular debris), and skeletal muscle showing numerous degenerate and regenerating myofibres with widespread necrosis accompanied by macrophages and scattered neutrophils. The serosal surface of the stomach, small intestine, large intestine, spleen and liver were thickened and hypercellular (peritonitis), changes which continued into the mesentery. The urinary bladder with numerous *N. caninum *cysts had widespread cell degeneration and necrosis of the detrusor muscle with numerous neutrophils and many macrophages. Necrosis and cellular debris in adrenal glands was associated with aggregations of *N. caninum*. Several small foci of degenerate myocardial fibres were infiltrated by macrophages and fewer neutrophils. The lungs had widespread collapse with markedly thickened alveolar walls infiltrated by many large macrophages and low to moderate numbers of neutrophils. In contrast to studies on gerbils and other rodents, our histopathological findings were not predominantly in the brain (malacia in cortex, meningoencephalitis), and we did not detect bronchopneumonia, which is characterised mainly by neutrophils [8]. The pancreas, adrenal gland or the urinary bladder have not been reported as sites of *N. caninum *infection in any host studied previously [4,8]. In an Australian context, the presence of large numbers of *N. caninum *cysts in dunnarts resembles findings of unusual cases of disseminated toxoplasmosis in magpie geese, which was dominated by *Toxoplasma gondii *cysts in liver and lesions in adrenal gland and pancreas associated with the parasite [26]. The myocardial changes detected in dunnarts are similar to those reported in toxoplasmosis in koalas [27].

All animals infected with a dose of 10^4 ^*N. caninum *tachyzoites (*n *= 3) survived, and their night activity, food consumption and water intake were comparable to the negative control animal (*n *= 1). Oil Red O staining of cryosectioned tails demonstrated fat deposits in the tail similar to controls (*P *= 0.82, 28 dpi, Figures 2c and 2d). Two of the three infected animals seroconverted as determined by cELISA (Table 1). PCR on DNA isolated from brain, lung and liver was negative for *N. caninum *(Figure 2b) and IHC staining was deemed negative in all tissues examined. No histological lesions were detected in any infected animal. At 10 dpi the immunosuppressed positive control animal suddenly developed severe paresis of all four limbs and reduced appetite (50% of the food remained unconsumed, probably due to immobility). Bodyweight reduced by 12% and the tail diameter reduced from 4.3 mm to 3.8 mm. This animal was euthanised at 11 dpi due to the sudden onset and severity of the clinical signs. PCR on DNA isolated from brain, lung, spleen, liver and blood was negative for *N. caninum*. Histopathology indicated minor changes in the spleen, urinary bladder, kidney and mesenteric lymph nodes consistent with per acute onset. Molecular techniques did not reveal the presence of *N. caninum *in examined tissues or blood, despite the animal having been inoculated intraperitoneally with 10^4 ^*N. caninum *tachyzoites and immunosuppressed with methylprednisolone acetate. Gross pathology did not reveal peritonitis (due to administration of *N. caninum*) or inflammation at the site of methylprednisolone acetate injection and all organs appeared normal compared to the control PBS-inoculated animals. The clinical findings resembled those in experiment B, and despite being negative for *N. caninum *by PCR, the immunosuppressed control dunnart is deemed to have succumbed to *N. caninum*. Due to a yet unknown host-parasite response in dunnarts prior to 13 dpi there is an apparent absence of indicative histopathological findings; an improved understanding of the pathogenesis of infection with *N. caninum *requires further experimentation. A lack of sufficient sensitivity of the molecular test cannot be excluded; PCR targeting Nc5 has been shown to be insufficiently sensitive during early stages of acute neosporosis in gerbils [6]. When gerbils (*M. unguiculatus*) were inoculated intraperitoneally with *N. caninum *(NC-Kr2) tachyzoites, parasite DNA was first detected on day 6 in DNA from liver or spleen using PCR and only from day 8 onwards throughout the body despite histologically confirmed focal miliary hepatitis being present after 1 day post-inoculation [6].

Following the tachyzoite experiments, we evaluated the infectivity of *N. caninum *oocysts and obtained material from a naturally shedding dog (dingo hybrid) from an Aboriginal community in the Tanami Desert, Northern Territory, Australia. The identity of *N. caninum *oocysts was confirmed by sequencing of ITS1 rDNA, [GenBank: HQ873010].

The two dunnarts fed *N. caninum *oocysts did not show clinical signs of neosporosis by the termination of the experiment and did not seroconvert to *N. caninum *(46 dpi). Using IHC, round cysts with zoites of *N. caninum *were identified in the spleen of one animal (E1, Table 1). The presence of *N. caninum *DNA was detected only in the brain of one animal using Nc5 primers (E2, Figure 2c). The presence of *N. caninum *was confirmed in brain DNA (E2) by amplification of *N. caninum *MS10 microsatellite using nested PCR [19,20]. Amplicons of MS10 from the brain DNA (E2) and from the DNA isolated from the oocysts used for inoculation were cloned and sequenced at AGRF (Westmead, Australia). Six and four MS10 genotypes were found within the DNA from *N. caninum *oocysts and dunnart's brain, respectively ([GenBank: HQ873011-HQ873034], Table 2, Additional file 3). Of eight unique MS10 genotypes found in the dunnart brain, two matched those in the GenBank™, EMBL and DDBJ databases, the NC-Nowra genotype was found in both DNAs and NC-GER1 found only in the oocysts' DNA (Table 2). It is possible that only certain genotypes will preferentially propagate within a given host. Moreover, contrary to the previously identified single MS10 genotypes reported from oocysts samples [3,19], the presence of multiple MS10 genotypes within the oocyst sample in this study suggests that *N. caninum *recombination in the definitive host may occur.

**Table 2:** Summary of *Neospora caninum *unique trinucleotide repeat pattern of MS10 microsatellite region.

Clones		#	Trinucleotide repeat pattern	Genotype
			
O-oocyst DNA	B-brain DNA			
O1, O2, O5, O6, O9, O12, O13, O14		8	(ACT)_6 _(AGA)_25 _(TGA)_8_	G1

O3		1	(ACT)_5 _(AGA)_24 _(TGA)_8_	G2

O4	B7	2	(ACT)_6 _(AGA)_22 _(TGA)_8 _*	G3

O7		1	(ACT)_6 _(AGA)_27 _(TGA)_7_	G4

O8, O10, 011	B1, B9	5	(ACT)_6 _(AGA)_24 _(TGA)_8_	G5

O15		1	(ACT)_6 _(AGA)_27 _(TGA)_8_	G6

	B2, B3, B4, B5, B8	5	(ACT)_6 _(AGA)_24 _(TGA)_9 _**	G7

	B6	1	(ACT)_6 _(AGA)_24 _(TGA)_7_	G8

*-matches NC-Nowra, [GenBank: GU128955].

**-matches NC-GER1, [GenBank: FJ824915].

#-number of clones.

The high susceptibility of marsupials to *N. caninum *parallels their susceptibility to a related pathogen *T. gondii *which also causes significant disease or death [28]. Existing evidence dictates that oocysts of *N. caninum *are produced in dogs and those of *T. gondii *in cats. However, it has been speculated that in Australia there may be a marsupial host capable of shedding *T. gondii *oocysts [28]. While we know that dingoes shed *N. caninum *oocysts, the life cycle of *N. caninum *in Australia is not fully understood [5]. Therefore, oocyst production by infected dunnarts was investigated. No oocysts resembling *N. caninum *(oocyst approximately 11-13 μm diameter containing two sporocysts) were detected using standard faecal flotation techniques during the experiment. Low levels of an unknown *Eimeria *sp. (oocyst approximately 20 μm diameter containing four sporocysts) were observed however.

Neosporosis in Australian native animals has not been previously studied. Our results provide the first experimental evidence that marsupials can be infected with *N. caninum*, thereby providing indirect support for the existence of a sylvatic life cycle. The susceptibility of the fat-tailed dunnart to *N. caninum *significantly advances our understanding of the disease beyond the recognised disease in farm and companion animals. It establishes the foundation for an investigation of the impact and costs of neosporosis to Australian wildlife. Combined with the recent identification of the dingo as a definitive host, high prevalence in feral mice, confirmation of abortion in a captive white rhinoceros due to neosporosis and knowledge of cerebral neosporosis in sheep, these results support of a domestic life cycle and a sylvatic life cycle between dingoes and small marsupials and rodents in Australia [5,29-31].

It is unknown whether *N. caninum *arrived with the dingo or with domestic or feral animals to Australia, but understanding the effects of *N. caninum *in marsupials and native rodents will be imperative for conserving their fragmented populations, already threatened by farm land encroachment. Experimental models like the fat-tailed dunnart will be essential to these discoveries. Furthermore, the extent and distribution of this disease in Australian marsupials is yet to be fully investigated. In the worst case scenario marsupials acquiring *N. caninum *may rapidly succumb to the disease at a high rate, meaning live wild marsupials and native rodents will not have been exposed to *N. caninum*, thereby providing a false indicator of the extent of the disease in Australia.

Under natural conditions we expect that carnivorous marsupials are infected by ingestion of *N. caninum *oocysts or tissue cysts. The unprecedented number of cysts widespread in the dunnart's musculature under experimental conditions using cell-cultured *N. caninum *tachyzoites improves our ability to produce infectious material for definitive host studies. Previously only beef offal infected with *N. caninum *was able to initiate often low oocyst production in canids [2,4]. In contrast to immunocompetent mice that are more resistant to *N. caninum *[10], dunnarts offer a new animal model where active neosporosis is dominated by tissue cysts presenting invaluable opportunities for further experimental study.

This work illustrates that laboratory bred dunnarts represent a novel model organism to study neosporosis and other infectious diseases that are recently established in or still exotic to Australia. Therefore dunnarts have the potential to be of significant value in understanding the costs of disease to wildlife that has not coevolved with the pathogen.

## Competing interests

The authors declare that they have no competing interests.

## Authors' contributions

JSK carried out the animal experiments, molecular detection studies and analysed the behaviour data. BM contributed the experimental animals and participated in the behavioural studies and data analysis. DSS and SL carried out the histopathology and immunohistochemistry analysis, and data interpretation. LH contributed histopathology data and analysis. AH contributed cryosection staining and analysis. SEA contributed parasite culture. JŠ, JSK, BM and JTE designed the experiments. JŠ conceived and coordinated the study, and wrote the manuscript. All authors read, approved and contributed to the final manuscript.

## Supplementary Material

Additional file 1 **Summary of food consumption for experimental animals**. Click here for file

Additional file 2 **Summary of body weights and tail diameters for experimental animals**. Click here for file

Additional file 3 **Sequence alignment of known *Neospora caninum *MS10 trinucleotide repeats**. Click here for file
