# Supplementary material for: Extensive production of Neospora caninum tissue cysts in a carnivorous marsupial succumbing to experimental neosporosis
Source: Vet Res. 2011 Jun 2;42(1):75. doi: 10.1186/1297-9716-42-75 (PMC3121614; doi:10.1186/1297-9716-42-75)

Additional file 1: Summary of food consumption for experimental animals

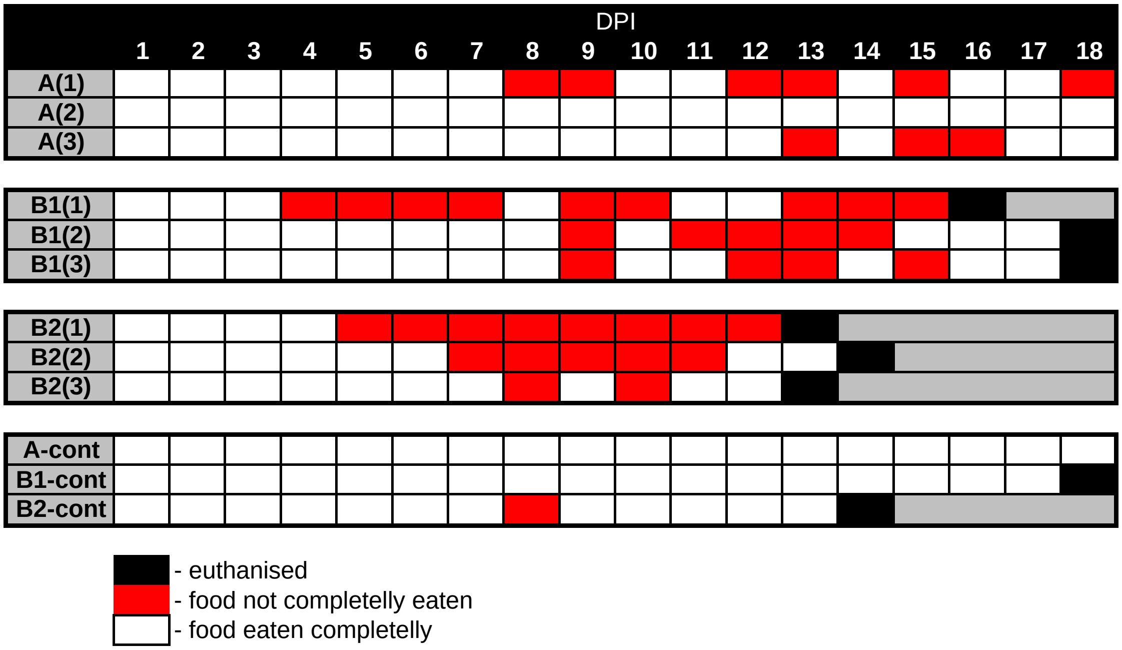

Supplement: Additional file 1 — Summary of food consumption for experimental animals. [file 1297-9716-42-75-S1.PDF]
