# Supplementary material for: Extensive production of Neospora caninum tissue cysts in a carnivorous marsupial succumbing to experimental neosporosis
Source: Vet Res. 2011 Jun 2;42(1):75. doi: 10.1186/1297-9716-42-75 (PMC3121614; doi:10.1186/1297-9716-42-75)
Supplement: Additional file 2 — Summary of body weights and tail diameters for experimental animals. [file 1297-9716-42-75-S2.PDF]

Additional file 2: Summary of body weights and tail diameters for experimental animals

Experiment Animal #	Euthanasia	Body weight (g)				Tail diameter (mm)			
		start	end	diff.	% diff.	start	end	diff.	% diff.
**A(1)**	28 dpi	16.3	16.0	**-0.3**	*-2%*	7.3	6.6	**-0.7**	*-10%*
**A(2)**	28 dpi	16.5	14.7	**-1.8**	*-11%*	5.1	4.7	**-0.4**	*-8%*
**A(3)**	28 dpi	12.8	12.4	**-0.4**	*-3%*	5.8	4.8	**-1.0**	*-17%*
**control -**	28 dpi	16.4	14.1	**-2.3**	*-14%*	4.9	5.0	**0.1**	*2%*
**control +**	11 dpi	16.5	14.7	**-1.8**	*-11%*	4.3	3.8	**-0.5**	*-12%*
**B1(1)**	16 dpi	15.4	15.2	**-0.2**	*-1%*	3.9	4.0	**0.1**	*3%*
**B1(2)**	18 dpi	17.1	15.9	**-1.2**	*-7%*	4.2	3.8	**-0.4**	*-10%*
**B1(3)**	18 dpi	13.0	14.0	**1.0**	*8%*	4.7	3.4	**-1.3**	*-28%*
**control -**	18 dpi	18.1	17.6	**-0.5**	*-3%*	4.3	6.4	**2.1**	*49%*
**B2(1)**	13 dpi	15.0	15.1	**0.1**	*1%*	4.8	3.3	**-1.5**	*-31%*
**B2(2)**	14 dpi	14.9	15.4	**0.5**	*3%*	4.7	2.9	**-1.8**	*-38%*
**B2(3)**	13 dpi	14.6	13.9	**-0.7**	*-5%*	3.6	2.7	**-0.9**	*-25%*
**control -**	14 dpi	16.2	15.9	**-0.3**	*-2%*	4.9	4.5	**-0.4**	*-8%*
**E1**	46 dpi	16.8	15.1	**-1.7**	*-10%*	5.5	4.1	**-1.4**	*-25%*
**E2**	46 dpi	15.4	15.1	**-0.3**	*-2%*	6.0	4.2	**-1.8**	*-30%*
